# Bis(1,2,3,4-tetra­hydro­quinoline-1-thio­carbon­yl) disulfide

**DOI:** 10.1107/S1600536812047320

**Published:** 2012-11-24

**Authors:** N. Srinivasan, S. Thirumaran, S. Selvanayagam

**Affiliations:** a Department of Chemistry, Annamalai University, Annamalainagar 608 002, India; b Department of Physics, Kalasalingam University, Krishnankoil 626 126, India

## Abstract

In the title compound, C_20_H_20_N_2_S_4_, the N-containing six-membered rings of the two tetra­hydro­quinoline moieties adopt half-chair conformations. Intra­molecular C—H⋯S hydrogen bonding stabilizes the mol­ecular structure. In the crystal, mol­ecules associate *via* weak C—H⋯π inter­actions.

## Related literature

For general background to the title compound, see: Von Deuten *et al.* (1980 ▶); Kumar *et al.* (1990 ▶); Fun *et al.* (2001 ▶). For preparation of the title compound, see: Garg *et al.* (1993 ▶). For related structures, see: Ivanov *et al.* (2003 ▶); Jian *et al.* (1999 ▶); Fun *et al.* (2001 ▶). For ring-puckering parameters, see: Nardelli (1983 ▶).
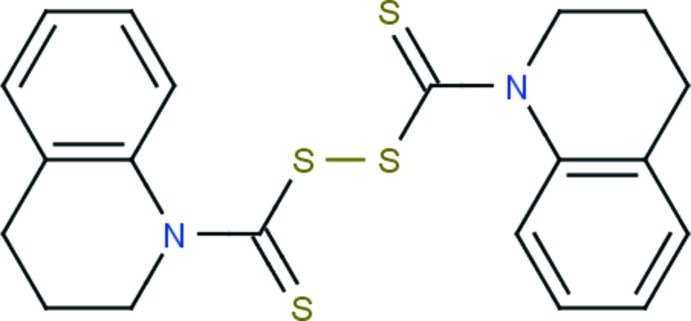


## Experimental

### 

#### Crystal data

C_20_H_20_N_2_S_4_
*M* *_r_* = 416.62
Monoclinic, 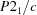

*a* = 8.1019 (4) Å
*b* = 20.3208 (11) Å
*c* = 12.3647 (6) Å
β = 104.371 (2)°
*V* = 1971.99 (17) Å^3^
*Z* = 4
Mo *K*α radiation
μ = 0.49 mm^−1^
*T* = 292 K
0.30 × 0.25 × 0.20 mm

#### Data collection

Bruker APEXII area-detector diffractometer
Absorption correction: multi-scan (*SADABS*; Bruker, 2008 ▶) *T*_min_ = 0.867, *T*_max_ = 0.909
44844 measured reflections
4941 independent reflections
4036 reflections with *I* > 2σ(*I*)
*R*_int_ = 0.031

#### Refinement

*R*[*F* ^2^ > 2σ(*F* ^2^)] = 0.038
*wR*(*F* ^2^) = 0.104
*S* = 1.03
4941 reflections
235 parameters
H-atom parameters constrained
Δρ_max_ = 0.53 e Å^−3^
Δρ_min_ = −0.26 e Å^−3^

### 

Data collection: *APEX2* (Bruker, 2008 ▶); cell refinement: *APEX2*; data reduction: *SAINT* (Bruker, 2008 ▶); program(s) used to solve structure: *SHELXS97* (Sheldrick, 2008 ▶); program(s) used to refine structure: *SHELXL97* (Sheldrick, 2008 ▶); molecular graphics: *ORTEP-3* (Farrugia, 1997 ▶) and *PLATON* (Spek, 2009 ▶); software used to prepare material for publication: *SHELXL97* and *PLATON*.

## Supplementary Material

Crystal structure: contains datablock(s) I, global. DOI: 10.1107/S1600536812047320/zq2186sup1.cif

Structure factors: contains datablock(s) I. DOI: 10.1107/S1600536812047320/zq2186Isup2.hkl

Supplementary material file. DOI: 10.1107/S1600536812047320/zq2186Isup3.cml

Additional supplementary materials:  crystallographic information; 3D view; checkCIF report

## Figures and Tables

**Table 1:** Hydrogen-bond geometry (Å, °) *Cg*1 is the centroid of the C4–C9 phenyl ring.

*D*—H⋯*A*	*D*—H	H⋯*A*	*D*⋯*A*	*D*—H⋯*A*
C12—H12*B*⋯S4	0.97	2.53	3.028 (2)	112
C18—H18⋯*Cg*1^i^		2.74	3.604 (2)	154

Symmetry code: (i) 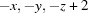.

**Fig. 1.**
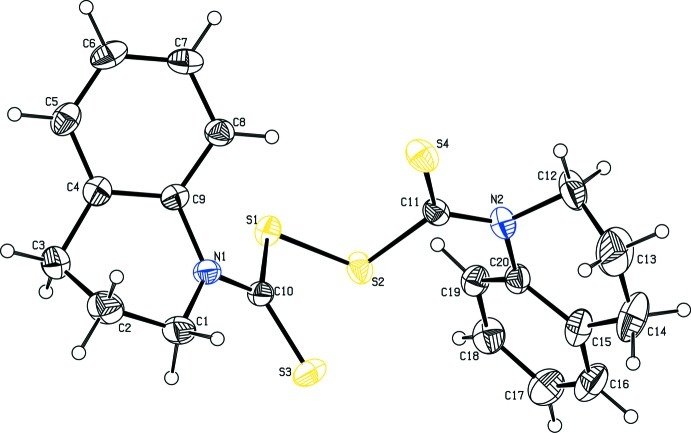
The molecular structure of the title compound, showing the atom-numbering scheme. Displacement ellipsoids are drawn at the 30% probability level

**Fig. 2.**
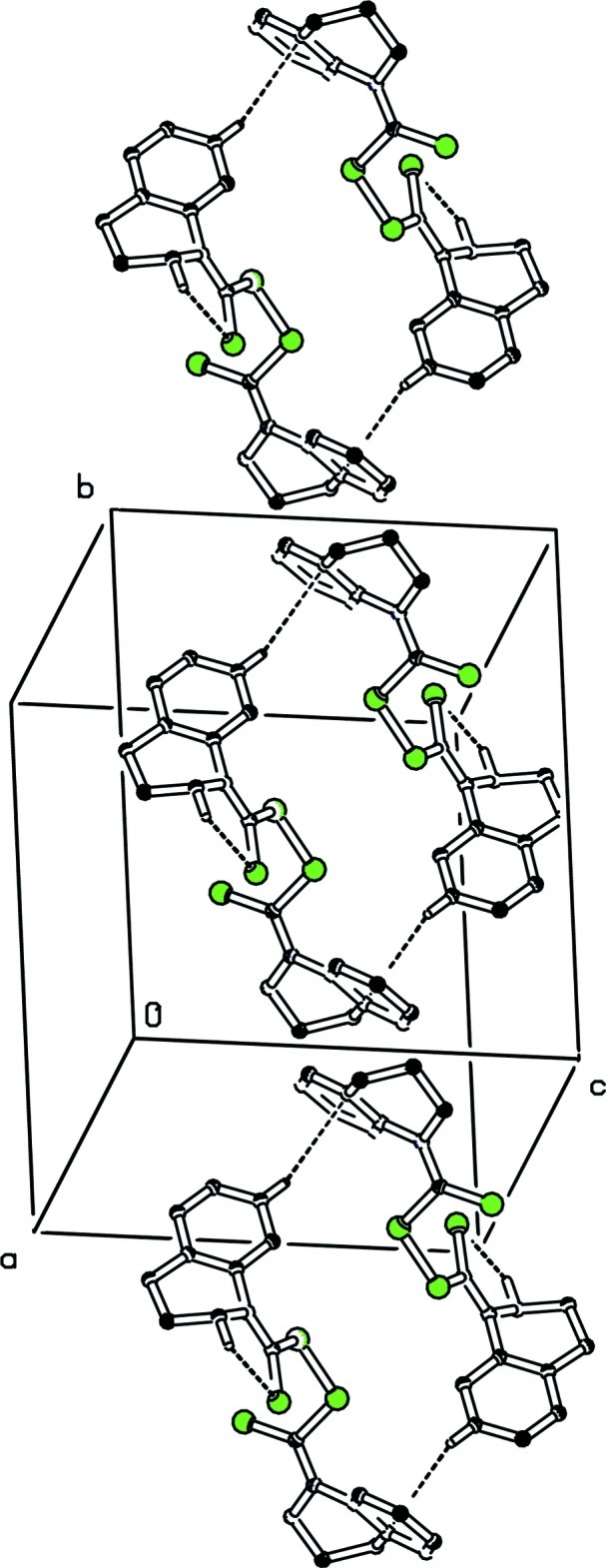
Molecular packing of the title compound, viewed along the a axis (H-bonds are shown as dashed lines). For the sake of clarity, H atoms, not involved in hydrogen bonds, have been omitted

## supplementary crystallographic information

### Comment

The bis(dialkylthiocarbomyl)disulfide compounds are the immediate oxidation products of dithiocarbamic acids and are known to be formed as one of the products during redox complexation reactions of dithiocarbamates with metal ions like Te^IV^, Se^IV^, La^III^ etc··· (Von Deuten *et al.*, 1980; Kumar *et al.*, 1990; Fun *et al.*, 2001). In the course of our investigations on the sodium salt of 1,2,3,4-tetrahydroquinolinecarbodithioate, we noticed the formation of bis(1,2,3,4-tetrahydroquinolinethiocarbomyl)disulphide. To study the structural features of this compound, we have undertaken its crystal structure determination and the results are presented here.

The X-ray study confirmed the molecular structure and atomic connectivity as illustrated in Fig. 1. The S—S bond distance of 1.9958 (6) Å is close to the related literature value (Ivanov *et al.*, 2003; Jian *et al.*, 1999; Fun *et al.*, 2001). Two sets of significantly different C—S distances are observed and these distances are clearly corresponding to single and double bonded C—S distances. The two C=S bonds are *trans* to each other. The N-containing six membered rings of the two tetrahydroquinoline moieties have a half-chair conformation with the lowest asymmetry parameters of ΔC_2_(N1-C9) = 0.038 (1)° and ΔC_2_(C12-N2) = 0.080 (1)° (Nardelli, 1983).

The molecular structure is influenced by an intramolecular C—H···S hydrogen bond (Fig. 2 and Table 1). In addition, intermolecular C—H···π interactions are observed with H18···Cg1^i^ = 2.74 Å, C18—H18···Cg1^i^ = 154°, and C18···Cg1^i^ = 3.604 (2) Å [Cg1 is the centroid of the phenyl ring (C4-C9) and the symmetry operation *i* corresponds to -*x*, -*y*, 2-*z*] (Fig. 2).

### Experimental

Recrystallization of sodium 1,2,3,4-tetrahydroquinolinecarbodithioate (Garg *et al.*, 1993) from a chloroform solution yielded the title compound as pale yellow crystals.

### Refinement

H atoms were placed in idealized positions and allowed to ride on their parent atoms, with C—H distances of 0.93-0.97 Å, and *U*_iso_(H) = 1.2*U*_eq_(C) for H atoms.

### Crystal data

**Table:**

C_20_H_20_N_2_S_4_	*F*(000) = 872
*M**_r_* = 416.62	*D*_x_ = 1.403 Mg m^−^^3^
Monoclinic, *P*2_1_/*c*	Mo *K*α radiation, λ = 0.71073 Å
Hall symbol: -P 2ybc	Cell parameters from 8987 reflections
*a* = 8.1019 (4) Å	θ = 2.5–28.3°
*b* = 20.3208 (11) Å	µ = 0.49 mm^−^^1^
*c* = 12.3647 (6) Å	*T* = 292 K
β = 104.371 (2)°	Block, yellow
*V* = 1971.99 (17) Å^3^	0.30 × 0.25 × 0.20 mm
*Z* = 4	

### Data collection

**Table:**

Bruker APEXII area-detector diffractometer	4941 independent reflections
Radiation source: fine-focus sealed tube	4036 reflections with *I* > 2σ(*I*)
Graphite monochromator	*R*_int_ = 0.031
ω and φ scans	θ_max_ = 28.4°, θ_min_ = 2.0°
Absorption correction: multi-scan (*SADABS*; Bruker, 2008)	*h* = −10→10
*T*_min_ = 0.867, *T*_max_ = 0.909	*k* = −25→27
44844 measured reflections	*l* = −16→14

### Refinement

**Table:**

Refinement on *F*^2^	Primary atom site location: structure-invariant direct methods
Least-squares matrix: full	Secondary atom site location: difference Fourier map
*R*[*F*^2^ > 2σ(*F*^2^)] = 0.038	Hydrogen site location: inferred from neighbouring sites
*wR*(*F*^2^) = 0.104	H-atom parameters constrained
*S* = 1.03	*w* = 1/[σ^2^(*F*_o_^2^) + (0.0495*P*)^2^ + 0.8147*P*] where *P* = (*F*_o_^2^ + 2*F*_c_^2^)/3
4941 reflections	(Δ/σ)_max_ = 0.001
235 parameters	Δρ_max_ = 0.53 e Å^−^^3^
0 restraints	Δρ_min_ = −0.26 e Å^−^^3^

### Special details

**Table:**

Geometry. All esds (except the esd in the dihedral angle between two l.s. planes) are estimated using the full covariance matrix. The cell esds are taken into account individually in the estimation of esds in distances, angles and torsion angles; correlations between esds in cell parameters are only used when they are defined by crystal symmetry. An approximate (isotropic) treatment of cell esds is used for estimating esds involving l.s. planes.
Refinement. Refinement of F^2^ against ALL reflections. The weighted R-factor wR and goodness of fit S are based on F^2^, conventional R-factors R are based on F, with F set to zero for negative F^2^. The threshold expression of F^2^ > 2sigma(F^2^) is used only for calculating R-factors(gt) etc. and is not relevant to the choice of reflections for refinement. R-factors based on F^2^ are statistically about twice as large as those based on F, and R- factors based on ALL data will be even larger.

### Fractional atomic coordinates and isotropic or equivalent isotropic displacement parameters (Å^2^)

**Table:**

	*x*	*y*	*z*	*U*_iso_*/*U*_eq_	
C1	0.7070 (2)	0.12620 (11)	0.98073 (17)	0.0529 (5)	
H1A	0.7149	0.1479	0.9124	0.064*	
H1B	0.7356	0.0802	0.9749	0.064*	
C2	0.8343 (3)	0.15668 (13)	1.0777 (2)	0.0640 (6)	
H2A	0.8437	0.2034	1.0642	0.077*	
H2B	0.9453	0.1369	1.0842	0.077*	
C3	0.7814 (3)	0.14684 (12)	1.18582 (18)	0.0585 (5)	
H3A	0.7667	0.1004	1.1988	0.070*	
H3B	0.8674	0.1644	1.2482	0.070*	
C4	0.6170 (2)	0.18262 (8)	1.17373 (15)	0.0425 (4)	
C5	0.5862 (3)	0.22676 (9)	1.25173 (16)	0.0510 (5)	
H5	0.6677	0.2324	1.3188	0.061*	
C6	0.4374 (3)	0.26245 (9)	1.23197 (18)	0.0555 (5)	
H6	0.4168	0.2904	1.2867	0.067*	
C7	0.3195 (3)	0.25670 (9)	1.13155 (18)	0.0520 (5)	
H7	0.2192	0.2810	1.1182	0.062*	
C8	0.3489 (2)	0.21484 (8)	1.04965 (16)	0.0437 (4)	
H8	0.2719	0.2127	0.9800	0.052*	
C9	0.4944 (2)	0.17619 (8)	1.07291 (13)	0.0367 (3)	
C10	0.4118 (2)	0.09180 (8)	0.92624 (13)	0.0383 (3)	
C11	−0.0542 (2)	0.09836 (8)	0.76717 (14)	0.0380 (3)	
C12	−0.2722 (3)	0.12260 (12)	0.59730 (19)	0.0682 (6)	
H12A	−0.3939	0.1145	0.5813	0.082*	
H12B	−0.2508	0.1677	0.6225	0.082*	
C13	−0.2106 (5)	0.11167 (18)	0.4968 (2)	0.1054 (11)	
H13A	−0.0911	0.1237	0.5127	0.126*	
H13B	−0.2724	0.1405	0.4380	0.126*	
C14	−0.2300 (5)	0.04240 (16)	0.4557 (2)	0.0900 (9)	
H14A	−0.1377	0.0325	0.4211	0.108*	
H14B	−0.3359	0.0389	0.3984	0.108*	
C15	−0.2305 (3)	−0.00897 (12)	0.54424 (17)	0.0596 (5)	
C16	−0.2603 (4)	−0.07527 (14)	0.5177 (2)	0.0752 (7)	
H16	−0.2693	−0.0890	0.4448	0.090*	
C17	−0.2768 (3)	−0.12063 (13)	0.5960 (2)	0.0734 (7)	
H17	−0.2927	−0.1648	0.5764	0.088*	
C18	−0.2701 (3)	−0.10132 (11)	0.70323 (19)	0.0586 (5)	
H18	−0.2844	−0.1320	0.7559	0.070*	
C19	−0.2420 (2)	−0.03635 (9)	0.73215 (16)	0.0455 (4)	
H19	−0.2413	−0.0227	0.8040	0.055*	
C20	−0.2147 (2)	0.00878 (9)	0.65492 (14)	0.0422 (4)	
N1	0.52917 (18)	0.13106 (7)	0.99185 (12)	0.0385 (3)	
N2	−0.18060 (19)	0.07682 (7)	0.68409 (13)	0.0444 (3)	
S1	0.22177 (6)	0.08105 (2)	0.97636 (3)	0.04264 (12)	
S2	0.06107 (6)	0.03269 (2)	0.85363 (4)	0.04694 (13)	
S3	0.44036 (8)	0.05131 (3)	0.81800 (4)	0.06045 (16)	
S4	0.00011 (7)	0.17623 (2)	0.79093 (5)	0.05336 (14)	

### Atomic displacement parameters (Å^2^)

**Table:**

	*U*^11^	*U*^22^	*U*^33^	*U*^12^	*U*^13^	*U*^23^
C1	0.0466 (10)	0.0613 (12)	0.0562 (11)	0.0073 (9)	0.0229 (9)	−0.0019 (9)
C2	0.0405 (10)	0.0747 (15)	0.0793 (15)	−0.0006 (10)	0.0196 (10)	−0.0060 (12)
C3	0.0498 (11)	0.0644 (13)	0.0549 (11)	0.0057 (9)	0.0009 (9)	−0.0048 (10)
C4	0.0478 (9)	0.0367 (8)	0.0431 (9)	−0.0052 (7)	0.0116 (7)	0.0007 (7)
C5	0.0648 (12)	0.0434 (10)	0.0436 (10)	−0.0123 (9)	0.0111 (9)	−0.0067 (8)
C6	0.0783 (14)	0.0384 (9)	0.0570 (12)	−0.0083 (9)	0.0304 (11)	−0.0139 (8)
C7	0.0597 (11)	0.0366 (9)	0.0651 (12)	0.0079 (8)	0.0258 (10)	−0.0026 (8)
C8	0.0490 (10)	0.0371 (9)	0.0460 (9)	0.0039 (7)	0.0135 (8)	0.0008 (7)
C9	0.0442 (9)	0.0309 (7)	0.0377 (8)	−0.0020 (6)	0.0155 (7)	0.0003 (6)
C10	0.0448 (9)	0.0364 (8)	0.0343 (8)	0.0076 (7)	0.0111 (7)	0.0011 (6)
C11	0.0365 (8)	0.0398 (8)	0.0393 (8)	0.0039 (6)	0.0125 (7)	0.0081 (7)
C12	0.0667 (14)	0.0599 (13)	0.0633 (13)	0.0076 (11)	−0.0118 (11)	0.0170 (11)
C13	0.150 (3)	0.102 (2)	0.0621 (16)	0.012 (2)	0.0215 (18)	0.0363 (16)
C14	0.119 (2)	0.112 (2)	0.0363 (11)	−0.0270 (19)	0.0133 (13)	0.0061 (13)
C15	0.0585 (12)	0.0782 (15)	0.0382 (10)	−0.0094 (11)	0.0045 (8)	−0.0036 (9)
C16	0.0833 (17)	0.0879 (18)	0.0507 (13)	−0.0143 (14)	0.0095 (12)	−0.0266 (12)
C17	0.0784 (16)	0.0589 (13)	0.0758 (16)	−0.0134 (12)	0.0056 (13)	−0.0232 (12)
C18	0.0581 (12)	0.0505 (11)	0.0625 (12)	−0.0118 (9)	0.0060 (10)	−0.0009 (9)
C19	0.0436 (9)	0.0501 (10)	0.0423 (9)	−0.0058 (8)	0.0099 (7)	−0.0012 (8)
C20	0.0357 (8)	0.0508 (10)	0.0376 (8)	−0.0013 (7)	0.0046 (7)	−0.0003 (7)
N1	0.0401 (7)	0.0385 (7)	0.0389 (7)	0.0036 (6)	0.0134 (6)	−0.0019 (6)
N2	0.0421 (8)	0.0454 (8)	0.0421 (8)	0.0009 (6)	0.0035 (6)	0.0090 (6)
S1	0.0451 (2)	0.0481 (2)	0.0342 (2)	−0.00396 (18)	0.00894 (17)	0.00092 (17)
S2	0.0513 (3)	0.0346 (2)	0.0467 (2)	−0.00014 (18)	−0.00342 (19)	0.00393 (17)
S3	0.0659 (3)	0.0696 (3)	0.0481 (3)	0.0074 (3)	0.0184 (2)	−0.0206 (2)
S4	0.0521 (3)	0.0362 (2)	0.0681 (3)	0.00046 (19)	0.0078 (2)	0.0086 (2)

### Geometric parameters (Å, º)

**Table:**

C1—N1	1.484 (2)	C11—S4	1.6491 (18)
C1—C2	1.508 (3)	C11—S2	1.8167 (16)
C1—H1A	0.9700	C12—C13	1.467 (4)
C1—H1B	0.9700	C12—N2	1.474 (2)
C2—C3	1.515 (3)	C12—H12A	0.9700
C2—H2A	0.9700	C12—H12B	0.9700
C2—H2B	0.9700	C13—C14	1.492 (5)
C3—C4	1.492 (3)	C13—H13A	0.9700
C3—H3A	0.9700	C13—H13B	0.9700
C3—H3B	0.9700	C14—C15	1.514 (3)
C4—C5	1.384 (3)	C14—H14A	0.9700
C4—C9	1.394 (2)	C14—H14B	0.9700
C5—C6	1.376 (3)	C15—C20	1.390 (3)
C5—H5	0.9300	C15—C16	1.393 (4)
C6—C7	1.371 (3)	C16—C17	1.367 (4)
C6—H6	0.9300	C16—H16	0.9300
C7—C8	1.388 (3)	C17—C18	1.371 (3)
C7—H7	0.9300	C17—H17	0.9300
C8—C9	1.386 (2)	C18—C19	1.372 (3)
C8—H8	0.9300	C18—H18	0.9300
C9—N1	1.437 (2)	C19—C20	1.381 (3)
C10—N1	1.347 (2)	C19—H19	0.9300
C10—S3	1.6355 (17)	C20—N2	1.438 (2)
C10—S1	1.8102 (18)	S1—S2	1.9958 (6)
C11—N2	1.332 (2)		
			
N1—C1—C2	112.79 (16)	C13—C12—H12A	110.2
N1—C1—H1A	109.0	N2—C12—H12A	110.2
C2—C1—H1A	109.0	C13—C12—H12B	110.2
N1—C1—H1B	109.0	N2—C12—H12B	110.2
C2—C1—H1B	109.0	H12A—C12—H12B	108.5
H1A—C1—H1B	107.8	C12—C13—C14	113.7 (3)
C1—C2—C3	111.09 (18)	C12—C13—H13A	108.8
C1—C2—H2A	109.4	C14—C13—H13A	108.8
C3—C2—H2A	109.4	C12—C13—H13B	108.8
C1—C2—H2B	109.4	C14—C13—H13B	108.8
C3—C2—H2B	109.4	H13A—C13—H13B	107.7
H2A—C2—H2B	108.0	C13—C14—C15	115.0 (2)
C4—C3—C2	106.76 (18)	C13—C14—H14A	108.5
C4—C3—H3A	110.4	C15—C14—H14A	108.5
C2—C3—H3A	110.4	C13—C14—H14B	108.5
C4—C3—H3B	110.4	C15—C14—H14B	108.5
C2—C3—H3B	110.4	H14A—C14—H14B	107.5
H3A—C3—H3B	108.6	C20—C15—C16	116.8 (2)
C5—C4—C9	118.17 (17)	C20—C15—C14	121.1 (2)
C5—C4—C3	123.85 (18)	C16—C15—C14	121.9 (2)
C9—C4—C3	117.65 (16)	C17—C16—C15	121.8 (2)
C6—C5—C4	121.30 (18)	C17—C16—H16	119.1
C6—C5—H5	119.3	C15—C16—H16	119.1
C4—C5—H5	119.3	C16—C17—C18	120.3 (2)
C7—C6—C5	119.92 (18)	C16—C17—H17	119.9
C7—C6—H6	120.0	C18—C17—H17	119.9
C5—C6—H6	120.0	C17—C18—C19	119.5 (2)
C6—C7—C8	120.40 (19)	C17—C18—H18	120.3
C6—C7—H7	119.8	C19—C18—H18	120.3
C8—C7—H7	119.8	C18—C19—C20	120.24 (19)
C9—C8—C7	119.15 (18)	C18—C19—H19	119.9
C9—C8—H8	120.4	C20—C19—H19	119.9
C7—C8—H8	120.4	C19—C20—C15	121.13 (19)
C8—C9—C4	120.86 (16)	C19—C20—N2	121.26 (16)
C8—C9—N1	121.37 (15)	C15—C20—N2	117.47 (17)
C4—C9—N1	117.66 (15)	C10—N1—C9	124.53 (14)
N1—C10—S3	124.66 (13)	C10—N1—C1	117.45 (14)
N1—C10—S1	113.48 (12)	C9—N1—C1	118.02 (14)
S3—C10—S1	121.63 (11)	C11—N2—C20	124.92 (14)
N2—C11—S4	124.95 (13)	C11—N2—C12	120.41 (16)
N2—C11—S2	113.40 (13)	C20—N2—C12	113.17 (15)
S4—C11—S2	121.64 (10)	C10—S1—S2	104.42 (6)
C13—C12—N2	107.8 (2)	C11—S2—S1	103.22 (6)
			
N1—C1—C2—C3	34.8 (3)	C16—C15—C20—N2	178.84 (19)
C1—C2—C3—C4	−63.3 (2)	C14—C15—C20—N2	−6.2 (3)
C2—C3—C4—C5	−129.7 (2)	S3—C10—N1—C9	167.43 (13)
C2—C3—C4—C9	43.5 (2)	S1—C10—N1—C9	−18.1 (2)
C9—C4—C5—C6	1.4 (3)	S3—C10—N1—C1	−13.8 (2)
C3—C4—C5—C6	174.54 (19)	S1—C10—N1—C1	160.63 (13)
C4—C5—C6—C7	−2.8 (3)	C8—C9—N1—C10	−42.2 (2)
C5—C6—C7—C8	0.3 (3)	C4—C9—N1—C10	141.44 (17)
C6—C7—C8—C9	3.6 (3)	C8—C9—N1—C1	139.04 (17)
C7—C8—C9—C4	−5.0 (3)	C4—C9—N1—C1	−37.3 (2)
C7—C8—C9—N1	178.73 (16)	C2—C1—N1—C10	−163.64 (18)
C5—C4—C9—C8	2.6 (3)	C2—C1—N1—C9	15.2 (2)
C3—C4—C9—C8	−171.03 (17)	S4—C11—N2—C20	171.86 (14)
C5—C4—C9—N1	178.96 (15)	S2—C11—N2—C20	−6.8 (2)
C3—C4—C9—N1	5.4 (2)	S4—C11—N2—C12	6.8 (3)
N2—C12—C13—C14	56.7 (4)	S2—C11—N2—C12	−171.84 (16)
C12—C13—C14—C15	−26.7 (4)	C19—C20—N2—C11	56.4 (3)
C13—C14—C15—C20	0.4 (4)	C15—C20—N2—C11	−127.8 (2)
C13—C14—C15—C16	175.1 (3)	C19—C20—N2—C12	−137.60 (19)
C20—C15—C16—C17	1.3 (4)	C15—C20—N2—C12	38.2 (2)
C14—C15—C16—C17	−173.7 (3)	C13—C12—N2—C11	103.4 (3)
C15—C16—C17—C18	2.3 (4)	C13—C12—N2—C20	−63.3 (3)
C16—C17—C18—C19	−1.8 (4)	N1—C10—S1—S2	172.06 (11)
C17—C18—C19—C20	−2.3 (3)	S3—C10—S1—S2	−13.32 (12)
C18—C19—C20—C15	6.0 (3)	N2—C11—S2—S1	−174.41 (12)
C18—C19—C20—N2	−178.37 (17)	S4—C11—S2—S1	6.88 (12)
C16—C15—C20—C19	−5.4 (3)	C10—S1—S2—C11	−90.59 (8)
C14—C15—C20—C19	169.6 (2)		

### Hydrogen-bond geometry (Å, º)

Cg1 is the centroid of the C4–C9 phenyl ring.

**Table:**

*D*—H···*A*	*D*—H	H···*A*	*D*···*A*	*D*—H···*A*
C12—H12*B*···S4	0.97	2.53	3.028 (2)	112
C18—H18···*Cg*1^i^		2.74	3.604 (2)	154

Symmetry code: (i) −*x*, −*y*, −*z*+2.
